# Steady State–Hopf Mode Interactions at the Onset of Electroconvection in the Nematic Liquid Crystal Phase V

**DOI:** 10.3390/ijms12074488

**Published:** 2011-07-13

**Authors:** Gyanu Acharya, Gerhard Dangelmayr, James Gleeson, Iuliana Oprea

**Affiliations:** 1 Department of Physics, Kent State University, Kent, OH 44242, USA; E-Mails: gacharya@kent.edu (G.A.); gleeson@physics.kent.edu (J.G.); 2 Department of Mathematics, Colorado State University, Fort Collins, CO 80523, USA; E-Mail: gerhard@math.colostate.edu

**Keywords:** nematic liquid crystals, electrohydrodynamic convection, mode interactions, pattern formation, amplitude equations

## Abstract

We report on a new mode interaction found in electroconvection experiments on the nematic liquid crystal mixture Phase V in planar geometry. The mode interaction (codimension two) point occurs at a critical value of the frequency of the driving AC voltage. For frequencies below this value the primary pattern-forming instability at the onset voltage is an oblique stationary instability involving oblique rolls, and above this value it is an oscillatory instability giving rise to normal traveling rolls (oriented perpendicular to and traveling in the director direction). The transition has been confirmed by measuring the roll angle and the dominant frequency of the time series, as both quantities exhibit a discontinuous jump across zero when the AC frequency is varied near threshold. The globally coupled system of Ginzburg–Landau equations that qualitatively describe this mode interaction is constructed, and the resulting normal form, in which slow spatial variations of the mode amplitudes are ignored, is analyzed. This analysis shows that the Ginzburg–Landau system provides the adequate theoretical description for the experimentally observed phenomenon. The experimentally observed patterns at and higher above the onset allow us to narrow down the range of the parameters in the normal form.

## 1. Introduction

Electroconvection in nematic liquid crystals is a physical system that serves as a testing ground for experimental studies and theoretical predictions of pattern formation in spatially extended anisotropic systems [1–5]. Its advantages over other pattern forming systems are the high aspect ratio (~10^3^), short time scales, and the adjustable control parameters (amplitude and frequency of the applied voltage). For electroconvection, the nematic is sandwiched between two glass plates, and an AC voltage is applied across the electrode plates. Above a critical value of the applied voltage, an electrohydrodynamic instability combined with a transition from the uniform state to a variety of patterns can occur, including stationary and traveling rolls as well as more complex spatiotemporal structures like worms, defects and spatiotemporal chaos [1,6–10].

Some of the patterns observed near the onset can be described by the standard model [5,11], which combines the theory of Ericksen and Leslie for an electrically conducting anisotropic fluid with the quasistatic Maxwell equations under the assumption of an ohmic charge conduction in the liquid crystal. Even with extra flexoelectric term, the standard model always predicts a stationary instability leading to stationary rolls, so it does not exhibit any oscillatory instability giving rise to the traveling wave patterns frequently observed near the onset in a variety of nematics like MBBA and Phase V [7,12,13], and I52 [14]. The weak electrolyte model [15], an extension of the standard model, in which a slow dissociation-recombination process of the charge carrying ions is taken into account and the ohmic behavior is replaced by the dynamics of two species of oppositely charged mobile ions, provides a basis for understanding the Hopf bifurcation that predicts the traveling wave patterns observed experimentally [7,16]. The weak electrolyte model can show an oscillatory instability as well as a stationary instability at the onset, and both types of instabilities can lead to oblique or normal rolls depending on the parameters, thus allowing for the occurrence of steady-oscillatory mode interactions.

A number of experimental and analytical studies of steady-oscillatory (Hopf) mode interactions in isotropic pattern forming systems, like Taylor–Couette flow, Rayleigh–Bénard convection, and convection in binary mixtures have been reported in the literature (see, e.g., [17–21] and references therein). There are no similar results in the literature for steady state-Hopf mode interactions in anisotropic systems. In this paper we report and analyze complex spatiotemporal patterns recorded in the electroconvection of the nematic mixture Phase V for AC voltages slightly above the onset value, which are dominated by normal oscillatory/oblique steady-state mode interaction. We observed, for two cells of almost the same thickness, oblique stationary modes at lower frequencies and normal traveling modes at higher frequencies, as well as a jump in the Hopf frequency from zero (stationary state) to over 20 rad/sec (normal traveling rolls) as the driving frequency is increased above a critical value.

The mode interaction point is identified with this critical value of the AC frequency along with the associated electroconvective threshold voltage. Since two parameters are fixed, this mode interaction point marks a codimension-two bifurcation point, which is rather different from the well known Lifshitz point. At the Lifshitz point a *continuous* transition from normal to oblique rolls of the *same type* (either stationary of traveling) occurs, whereas the codimension-two point reported in this paper marks a transition from oblique stationary to normal traveling rolls combined with a discontinuity in the Hopf frequency as well as wave numbers and roll angle.

The experimentally observed behavior can be understood through the amplitude equations that follow from a governing system of partial differential equations for anisotropic systems in a weakly nonlinear analysis. In this paper we do not pursue this analysis for the equations for nematic electroconvection, but instead use symmetry considerations to set up the generic form of the system of globally coupled Ginzburg–Landau equations that describes the patterns observable near the mode interaction point. The analysis of the resulting normal form, in which slow variations of the amplitudes are ignored, in a range of parameters consistent with the experiments, provides a possible theoretical scenario corresponding to the experimentally observed phenomena.

We report here, for the first time in the literature, experimental evidence of a steady oblique–normal traveling mode interaction point. Our experiments have been performed in the conduction regime, below the cutoff value of the driving AC frequency. In a previous study [22], a phase diagram for a Phase V sample sandwiched in a “channel” (thin cell in one direction) is presented in which a transition from traveling to steady convection occurs at the cutoff frequency. This transition also corresponds to a steady-traveling mode interaction, however it does not involve interaction of rolls oriented in different directions. Moreover, due to the small aspect ratio of the channel, the amplitude description involves 1D Ginzburg–Landau equations, and both traveling and steady rolls are normal rolls (of different wavelengths). In contrast, the amplitude equations needed to capture our experimental situation are 2D anisotropic Ginzburg–Landau equations.

The outline of the paper is as follows. In Section 2 we describe the experimental setup and the observed electroconvective patterns. In Section 3 we introduce a system of globally coupled Ginzburg–Landau amplitude equations that captures near-onset patterns in the neighborhood of the mode interaction point, and perform a bifurcation analysis of the associated normal form. Section 4 is devoted to a discussion of the results and concluding remarks.

## 2. Experiments

### 2.1. Experimental Set Up

The experiments were conducted with the nematic liquid crystal (NLC) Phase V [7,23], a mixture of 65 wt% 4-butyl-4′-methoxyazoxybenzene and 35 wt% 4-ethyl-4′-methoxyazoxybenzene with nematic range between −5 °C and 75 °C. The NLC was sandwiched in a ready made sample cell fabricated by E.H.C. Co., Tokyo, Japan (EHC-cell) with flat transparent electrodes, which are rubbed to produce planar alignment of the director in a fixed (*x*) direction. The electrodes are made of indium tin oxide coated borosilicate glass plates and provide an active lateral area of *A* = 10 × 10mm^2^. Outside of the active area, there is no conductive coating and hence no electrical field is present. In this parallel-plate-capacitor geometry, the electrodes are separated by a vertical distance, *d*, which was measured interferometrically. The electrical contact between the plates and the hookup wires was created using silver-laden epoxy.

Usually a small amount of dopant (tetrabutylammium bromide) is added to the NLC to increase the conductivity. For our sample the conductivity was sufficient to observe the desired electroconvection states, thus the experiments have been conducted without added dopant. The conductivity varies between individual cells with temperature, AC frequency, and time elapsed, and threshold voltages vary accordingly. To obtain the conductivity, *σ*⊥, and the dielectric permittivity, *ε*⊥, perpendicular to the director, we measured the conductance (*G*⊥ = *Aσ*⊥/*d*) and the capacitance (*C*⊥ = *Aε*_0_*ε*⊥/*d*) using a capacitance bridge.

The electroconvection apparatus consisted of a temperature-controlled hot stage, electronics for applying the AC voltage, and a shadowgraph [24] apparatus for visualization. The cell was illuminated by polarized light with the polarization along the director, and the resulting shadowgraph images were monitored by a charge-coupled device camera mounted on the microscope, using a 10× objective. The frame grabber hooked up to the camera captures 8 bit grey scale images of size 480 × 640 pixels at a rate of up to 30 frames per second. The images recorded cover an area of 358.21 × 477.61 *μ*m^2^ from the active area of the cell. In order to remove inhomogeneities in the optical system, the raw images have been flat-field corrected using the background and dark frames of the system, see [25] for details.

The EHC experiments have been performed in two cells A and B of thicknesses

$$\begin{array}{ll} \text{cell A}: & d=10.63\pm 0.09\;\mu \text{m}\\ \text{cell B}: & d=10.07\pm 0.08\;\mu \text{m} \end{array}$$

The experiments in cell B were control experiments to confirm the reproducibility of the EHC-patterns observed in cell A. The electroconvection was driven by applying an AC voltage of frequency *f*_0_ (circular frequency *ω*_0_) and voltage *V* to the electrodes. The driving frequency was increased in certain steps adapted to the variation of the onset voltage *V_c_*. For our sample the dielectric anisotropy was negative, and the AC frequency was varied in the conduction regime, *i.e*., below the cutoff frequency which was calculated from measured and tabulated [7] material parameters using the formula given in [11].

To record near-onset patterns for a fixed AC frequency, we slowly increased *V* (0 < *ε* = *V*^2^/*V*^2^_c_ – 1 ≪ 1), waited a few minutes, and then captured a shadowgraph image and calculated its spatial power spectrum *S*(**k**), **k** = (*p*, *q*). Focusing was adjusted to enhance the dominant inner modes (first harmonics) of *S*(**k**). The sample stage was rotated to ensure that the peaks of *S*(**k**) were equidistant from the axes. Next, the stage was fixed for the whole experiment, and a short movie of 2048 frames was captured. To see changes in the patterns above the onset, for some frequencies the experiments have been repeated for higher values of *V*.

**Figure 1:**
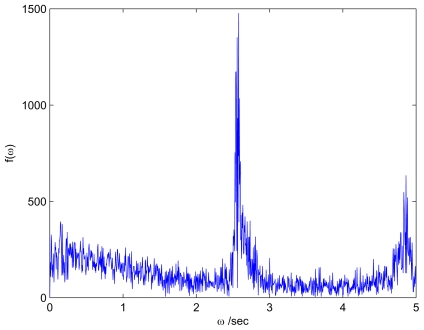
*P*(*ω*) for cell A at 35 °C and *f*_0_ = 350 Hz corresponding to *ω*_0_*τq* = 1.07, where *τq* is the charge relaxation time. The circular Hopf frequency is *ω_H_* = 16.21 radians/s (*f_H_* = 2.58 Hz).

To characterize the temporal behavior of the near-onset patterns, we computed the power spectrum, *P*(*ω*), of the time series of the central pixel value for each individual movie. A sharp maximum of *P*(*ω*)at a nonzero circular frequency *ω_H_* > 0 was considered as the indicator of traveling rolls, and the instability at the onset was identified with a Hopf bifurcation (HB) (Hopf frequency *f_H_* = *ω_H_*/2π). An example of *P*(*ω*) with *ω_H_* > 0 is shown in Figure 1. If the maximum occurred at *ω_H_* ≈ 0, we identified the instability with a steady state bifurcation (SSB). In our experiments normal traveling (NT) rolls were excited at Hopf bifurcations, and oblique stationary (OS) rolls at steady state bifurcations.

### 2.2. Results

**Figure 2:**
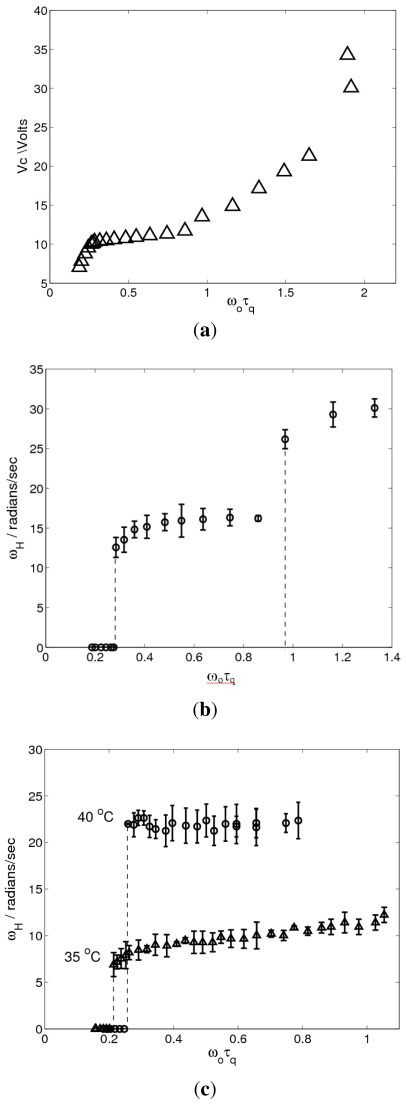
(**a**) Threshold curve *V_c_* and (**b**) circular frequency *ω_H_* versus *ω*_0_*τ_q_* for cell A at 35 °C. In (**c**) *ω_H_* is shown for cell B at 35 °C and 40 °C. The vertical dashed lines in (b) and (c) mark discontinuous jumps.

Figure 2(a) shows the onset voltage for cell A at 35 °C as function of the dimensionless driving frequency *ω*_0_*τ_q_*, where *τ_q_* = *ε*_0_*ε*⊥/*σ* ⊥ is the charge relaxation time. The variation of *ω_H_* in this range is displayed in Figure 2(b). While the threshold curve is continuous, we observe two discontinuous jumps in *ω_H_* which are marked by vertical dashed lines. The first jump is from *ω_H_* = 0 to *ω_H_* = 12.57 radians/s (*f_H_* = 2.0 Hz) and occurs at *f*_0_ = 92 Hz (*ω*_0_*τ_q_* = 0.28). This jump clearly corresponds to a transition from a steady state bifurcation to a Hopf bifurcation (Section 3). The origin of the second jump is unknown at present.

To test the reproducibility of the discontinuity in *ω_H_*, we have performed the same experiment in cell B at 35 °C and 40 °C. The variations of *ω_H_* with the driving frequency for these temperatures are shown in Figure 2(c). In both cases we can recognize again the jumps from *ω_H_* = 0 to nonzero value of *ω_H_* occurring at values of *ω*_0_*τ_q_* close to 0.28. The second discontinuity found in cell A is either not present or occurs at a higher value, outside of the frequency range studied for cell B, *i.e.*, at some *ω*_0_*τ_q_* > 1.

**Figure 3:**
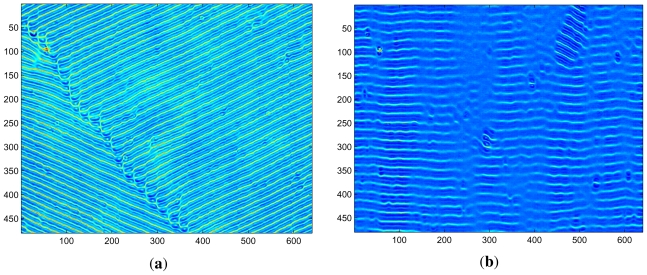
Snapshots of patterns in cell A at 35 °C for (**a**) *f*_0_ =90 Hz (*ω*_0_*τ_q_* =0.27), *V* =10.38V (*ε* = 0.05), and (b) *f*_0_ =95 Hz (*ω*_0_*τ_q_* =0.29), *V* =10.39V (*ε* = 0.016).

**Figure 4:**
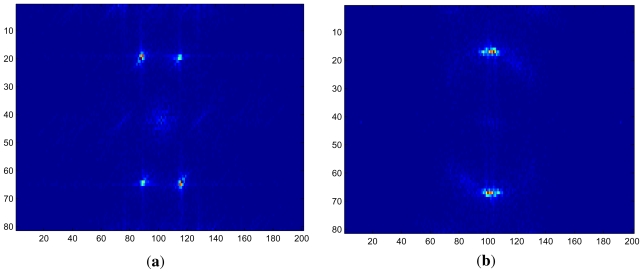
(**a**) and (**b**): Amplitude of Fourier transform of the images depicted in Figure 3(a), (b), respectively, into regions showing the dominant first harmonics.

In order to characterize the transition at the first jump, we studied the patterns created slightly above the onset for *ω*_0_*τ_q_* below and above the discontinuity in *ω_H_*. In Figure 3(a),(b), near-onset pattern snapshots are shown for *f*_0_ = 90 Hz (*ω*_0_*τ_q_* = 0.27) and *f*_0_ = 95 Hz (*ω*_0_*τ_q_* = 0.29), with voltages *V* = 10.38 V (*ε* = 0.05) and *V* = 10.39 V (*ε* = 0.016), respectively. The pattern in Figure 3(a) is stationary (which can be seen in the movie) and made up of oblique rolls, whereas the almost normal rolls apparent in (b) are traveling vertically upwards. The moduli of the Fourier transforms of the images shown in Figure 3(a),(b) are shown in Figure 4(a),(b). For both patterns, we can clearly recognize sharp peaks at the dominant (critical) wave numbers corresponding to oblique and normal rolls. The peaks at higher harmonics (not shown in Figure 4) are significantly smaller than the peaks at the first harmonics.

When the applied voltage in cell A at 35 °C at *f*_0_ = 95 Hz was increased further above threshold, we observed a stationary pattern made up of oblique rolls again (not shown here). In contrast, when *V* was further increased above threshold for *f*_0_ = 90 Hz, no qualitative change in the pattern dynamics was observed. Thus above the jump the near-onset patterns are NT rolls, but the OS rolls reappear when the voltage is increased further.

**Figure 5:**
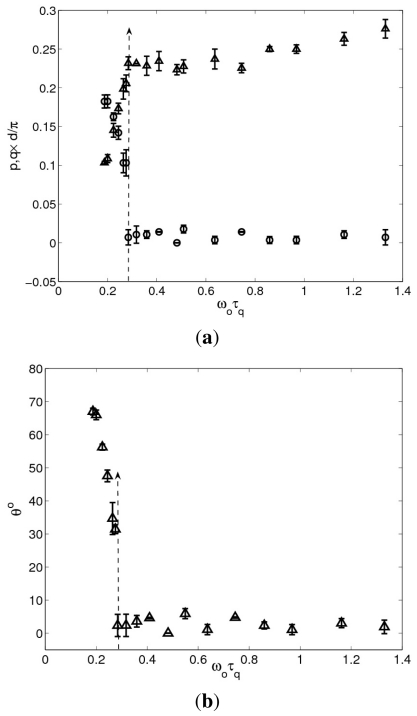
Variation of (**a**) the non-dimensionalized average wave numbers *<p> d/π* (up triangles) and *<q> d/π* (circles); and (**b**) the average roll angle *θ* with the normalized frequency in cell A at 35 °C. The dashed arrow differentiates between OS (left) and NT (right) rolls.

**Figure 6:**
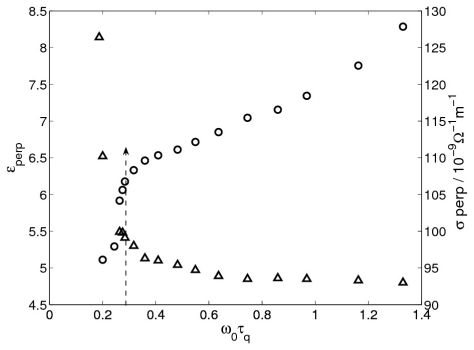
Variation of *ε* _⊥_ (up triangles) and *σ*_⊥_ (circles) with *ω*_0_*τ_q_* for cell A at 35 °C. The dashed arrow marks the critical *ω*_0_*τ_q_*-value separating the OS and NT regimes.

To confirm the transition from normal to oblique rolls at onset, we computed the average of the horizontal and vertical wave numbers,

$$<p>=\int pS_{a}(p,q)\;dpdq,\;\;\;<q>=\int qS_{a}(p,q)\;dpdq$$

where *S_a_*(*p, q*) is the average of *S*(*p, q*) over the first 100 frames, normalized such that ∫*S_a_*(*p, q*)*dpdq* = 1. The variations of these averages, and of the corresponding average roll angle,

$$\theta =\text{arctan}\frac{<p>}{<q>}$$

with *ω*_0_*τ_q_* are shown in Figure 5(a, b), respectively. In these plots we can see jumps in *<q>* and *θ* at *ω*_0_*τ_q_* ≈ 0.28 from nonzero to zero values, showing that there is a transition from oblique to normal rolls. Notice that in the oblique regime *<p>* and *<q>* show a relatively strong variation with the driving frequency, whereas in the normal regime there is little variation. In addition to this strong variation in the average wave numbers, there is also a strong variation of *σ*_⊥_ and *ε* _⊥_ (but not a discontinuity) in the oblique regime, see Figure 6.

## 3. Amplitude Equations and Bifurcation Diagrams

### 3.1. Codimension-two Mode Interactions

The observed coincidence of a stationary and an oscillatory instability is commonly referred to as a codimension-two mode interaction [26]. Codimension two here means that two parameters, in our case the threshold voltage and the AC frequency, have to be adjusted. This mode interaction can be explained by coincident minimal voltages on two neutral stability surfaces. The extended model for EHC in NLC, the weak electrolyte model [7,15,27], can show a Hopf as well as a stationary instability at the onset, and they both can lead to oblique or normal rolls depending on the parameters. In either case, the critical onset voltage is the minimum of a neutral stability surface in (*V, p*^2^*, q*^2^)-space, on which an eigenvalue of the linearized system is either zero (stationary case) or purely imaginary (oscillatory case).

Let us denote by *V_s_*(*p*^2^*, q*^2^*, ω*_0_) the stationary neutral stability surface with minimum *V_sc_*(*ω*_0_) = *V_s_*(*p*^2^*_sc_*, *q*^2^*_sc_*, *ω*_0_), and by *V_o_*(*p*^2^*, q*^2^*, ω*_0_) the oscillatory neutral stability surface with minimum *V_oc_*(*ω*_0_) = *V_o_*(*p*^2^*_oc_, q*^2^*_oc_, ω*_0_) and Hopf frequency *ω_H_*(*ω*_0_) at criticality. Our experiments suggest that *q_oc_*(*ω*_0_) = 0 since NT rolls are observed, and *V_oc_*(*ω*_0_) = *V_sc_*(*ω*_0_) at the critical value of the AC frequency (*ω*_0_*_c_τ_q_* = 0.28 for cell A at 35 °C). Moreover, *V_oc_*(*ω*_0_) *> V_sc_*(*ω*_0_) if *ω*_0_ *< ω*_0_*_c_* and *V_oc_*(*ω*_0_) *< V_sc_*(*ω*_0_) if *ω*_0_ *> ω*_0_*_c_*. Thus the critical wave numbers at onset are (*p_sc_*(*ω*_0_)*; q_sc_*(*ω*_0_)) for *ω*_0_ *< ω*_0_*_c_* and (*p_oc_*(*ω*_0_)*,* 0) for *ω*_0_ *> ω*_0_*_c_*. The codimension-two mode interaction point is given by fixing *ω*_0_ = *ω*_0_*_c_* and *V* = *V_c_*, with *V_c_* = *V_sc_*(*ω*_0_*_c_*) = *V_oc_*(*ω*_0_), and has frequency *ω_Hc_* = *ω_H_*(*ω*_0_*_c_*) associated with the NT rolls.

In general there should be no special relation between the location of the minima on the two neutral stability surfaces, thus a jump can be expected in both the horizontal and vertical critical wave numbers as we have found in the experiments. We note that the codimension-two mode interaction point described above is very different from the well-known Lifshitz point [3,4]. The main difference is that the Lifshitz point involves a single neutral stability surface (either the stationary or the oscillatory one) and there is a *continuous* transition from oblique to normal rolls, whereas the mode interaction reported here involves two different neutral stability surfaces and leads to jumps.

### 3.2. Globally Coupled Ginzburg–Landau Equations

We now describe the derivation of the system of globally coupled Ginzburg–Landau amplitude equations that captures near-onset patterns in a vicinity of the mode interaction point.

The weak electrolyte model (WEM) consists of partial differential equations derived from the Navier-Stokes equation for an anisotropic electrically conducting fluid, the conservation of charge, Poisson’s law, and a partial differential equation for the conductivity. The WEM equations are extremely complicated for a fully 3D numerical simulation, therefore a weakly nonlinear analysis at the onset is particularly useful. In this analysis, the patterns above threshold are represented as superposition of OS and NT modes in the form

(1) $$\begin{array}{l} u(t,x,y,z)=ɛ(Ae^{ip_{sc}x}U_{s+}(z)+Be^{-ip_{sc}x}U_{s-}(z))e^{iq_{sc}y}+\\ ɛ(Ce^{ip_{oc}x}U_{o+}(z)+De^{-ip_{oc}x}U_{o-}(z))e^{i\omega_{Hc}t}+\text{cc}+O({ɛ}^{2}) \end{array}$$

where u represents the field variables of the WEM (velocities, electric potential, director, conductivity, see [15,16,27,28]), *A*, *B* and *C*, *D* are slowly varying complex envelopes of the OS-rolls and the counter-propagating NT-rolls, respectively, *ɛ* is a small parameter, *ɛ*^2^ ~ |*V/V_c_* − 1|, U*_s±_* (*z*) and U*_o±_* (*z*) are vertical critical modes, and cc refers to the complex conjugate expression. All envelopes are functions of a slow time *T* = *ɛ*^2^*t* and slow space variables. Specifically, *A*, *B* depend on (*X, Y* ) = (*ɛx, ɛy*), and *C* and *D* depend on (*X*_+_*, Y*) and (*X*_−_*, Y*), respectively, where *X*_±_ = *ɛ*(*x* ± *υ_c_t*) and *υ_c_* is the critical group velocity derived from the oscillatory neutral stability surface at criticality, see [29,30].

The form of the system of globally coupled Ginzburg–Landau equations for the envelopes follows in a straightforward manner from symmetry considerations combined with a formal multiple scale expansion and an appropriate rescaling of the envelopes and the slow variables as (the subscript *T* denotes derivative with respect to the slow time *T*)

(2) $$\begin{array}{l} A_{T}=(\Lambda_{s}+D_{s}(\partial_{X},\partial_{Y})\;-|A{|}^{2}-\;a|B{|}^{2}+\;c<|C{|}^{2}>+\bar{c}<\;|D{|}^{2}>)A\\ B_{T}=(\Lambda_{s}+D_{s}(-\partial_{X},\partial_{Y})\;-|B{|}^{2}-\;a|A{|}^{2}+\;c<\;|D{|}^{2}>+\bar{c}<\;|C{|}^{2}>)B\\ C_{T}=(\Lambda_{o}+D_{o}(\partial_{X_{+}},\partial_{Y})-b_{1}|C{|}^{2}-\;b_{2}<\;|D{|}^{2}>+d<\;|A{|}^{2}+|B{|}^{2}>)C\\ D_{T}=(\Lambda_{o}+D_{o}(\partial_{X_{+}},\partial_{Y})-b_{1}|D{|}^{2}-\;b_{2}<\;|C{|}^{2}>+d<\;|A{|}^{2}+|B{|}^{2}>)D \end{array}$$

where *a* is real and *b*, *b*_1_, *b*_2_, *c*, *d* are complex coefficients. The diffusion operators in (2) are

(3) $$\begin{array}{l} D_{s}(\partial_{X},\partial_{Y})=\partial_{X}^{2}+2\delta \partial_{X}\partial_{Y}+\partial_{Y}^{2}\\ D_{o}(\partial_{X_{\pm }},\partial_{Y})=\alpha \partial_{X_{\pm }}^{2}+\beta \partial_{Y}^{2} \end{array}$$

with a further real coefficient *δ*, *δ*^2^ *<* 1, and complex coefficients *α*, *β* with positive real parts. The coefficients *υ_c_* and *a*, *b*_1_, *b*_2_, *c*, *d* are computable from the linear and the quadratic and cubic terms of the constitutive equations of the WEM, respectively [16]. Due to the different variables on which the envelopes depend, the Ginzburg–Landau Equations (2) contain global coupling terms involving spatial averages

$$<\;|F{|}^{2}>=\underset{L\to \infty }{\text{lim}}\frac{1}{2L}\int_{-L}^{L}|F(T,\xi ,Y){|}^{2}d\xi$$

where *F* represents any of the four envelopes and *ξ* = *X* if *F* = *A* or *B*, whereas *ξ* = *X*_+_ if *F* = *C* and *ξ* = *X*_−_ if *F* = *D*. The presence of these global coupling terms is due to the assumption of a finite group velocity and follows in a similar way as in the case of an oscillatory instability leading to oblique traveling rolls [29,30]. Finally, Λ*_s_* and Λ*_o_* are *O*(1) “unfolding parameters” describing deviations of *V* and *ω_o_* from the codimension-two point

(4) $$\Lambda_{s}=\lambda -b_{s}\mu ,\;\;\;\Lambda_{o}=a_{o}\lambda -b_{o}\mu$$

where

(5) $${ɛ}^{2}\lambda =\frac{V}{V_{c}}-1,\;\;\;{ɛ}^{2}\mu =\frac{\omega_{0}}{\omega_{0c}}-1$$

with a further real coefficient *b_s_* and complex coefficients *a_o_*, *b_o_*. The coefficient of *λ* in Λ*_s_* has been normalized to unity which can be achieved by a rescaling of *T*.

We note that the system (2) holds in the generic case when *p_sc_*/*p_oc_* is irrational. If this ratio is rational, additional coupling terms have to be included.

### 3.3. Normal Form and Bifurcation Diagrams

The Ginzburg–Landau system (2) provides the correct amplitude description for the type of instability considered, and results of numerical simulations of (2) will be described elsewhere. In this paper we confine ourselves to spatially uniform solutions and present bifurcation diagrams consistent with our experiments. Ignoring spatial variations of the envelopes leads to the following system of normal form equations

(6) $$\begin{array}{l} A_{T}=(\Lambda_{s}-|A{|}^{2}-\;a|B{|}^{2}+\;c|C{|}^{2}+\;\bar{c}|D{|}^{2})A\\ B_{T}=(\Lambda_{s}-|B{|}^{2}-\;a|A{|}^{2}+\;c|D{|}^{2}+\;\bar{c}|C{|}^{2})B\\ C_{T}=(\Lambda_{o}-b_{1}|C{|}^{2}-\;b_{2}|D{|}^{2}+\;d(|A{|}^{2}+|B{|}^{2}))C\\ D_{T}=(\Lambda_{o}-b_{1}|D{|}^{2}-\;b_{2}|C{|}^{2}+\;d(|A{|}^{2}+|B{|}^{2}))D \end{array}$$

In polar coordinates, *A* = *r_A_e^iϕA^*, *etc.*, the radial parts satisfy the following system of equations which is decoupled from the phases (we use subscripts *r* and *i* to denote real and imaginary parts)

(7) $$\begin{array}{l} r_{A,T}=(\Lambda_{s}-r_{A}^{2}-ar_{B}^{2}+c_{r}(r_{C}^{2}+r_{D}^{2}))r_{A}\\ r_{B,T}=(\Lambda_{s}-r_{B}^{2}-ar_{A}^{2}+c_{r}(r_{C}^{2}+r_{D}^{2}))r_{B}\\ r_{C,T}=(\Lambda_{or}-b_{1r}r_{C}^{2}-b_{2r}r_{D}^{2}+d_{r}(r_{A}^{2}+r_{B}^{2}))r_{C}\\ r_{D,T}=(\Lambda_{or}-b_{1r}r_{D}^{2}-b_{2r}r_{C}^{2}+d_{r}(r_{A}^{2}+r_{B}^{2}))r_{D} \end{array}$$

The basic (conduction) state corresponds to the trivial solution T : *A* = *B* = *C* = *D* = 0. The basic state is stable if Λ*_s_* *<* 0 and Λ*_or_* *<* 0. On Λ*_s_* = 0 two types of stationary “pure mode” solutions bifurcate from T: The OS-rolls satisfying *r_A_* = *R >* 0, *r_B_* = 0, *r_C_* = *r_D_* = 0, and stationary rectangle (SR) solutions which satisfy *r_A_* = *r_B_* = *R >* 0, *r_C_* = *r_D_* = 0. Both OS and SR are fixed points of (7) and (6) and only one of them can be stable in a region of the (Λ*_s_,* Λ*_or_*)-plane. The condition that OS is stable, as observed in our experiments, requires that

(8) a>1

At Λ*_or_* = 0 two types of oscillatory “pure mode” solutions bifurcate from T: NT-rolls satisfying *r_C_* = *R >* 0, *r_D_* = 0, *r_A_* = *r_B_* = 0, and standing wave (SW) solutions which satisfy *r_C_* = *r_D_* = *R >* 0, *r_A_* = *r_B_* = 0. These solutions are also fixed points of (7) but periodic solutions of (6), and only one of them can be stable. The condition that NT is stable requires that

(9) $$b_{2r}>b_{1r}>0$$

As common in mode interaction normal forms [26], in addition to the primary pure mode solutions, mixed mode solutions branch off the primary solutions in secondary bifurcations. These mixed mode solutions are also fixed points of (7) and are revealed as superpositions of a primary stationary solution and a primary oscillatory solution. Among the four possible mixed mode solutions, only the superposition of OS and NT is relevant in our case, and we refer to this solution as MM. The MM satisfies *r_A_* = *R_o_* *>* 0, *r_C_* = *R_n_* *>* 0, and *r_B_* = *r_D_* = 0.

The equations for OS, NT, and MM are

(10) OS:R2=ΛsNT:R2=Λor/b1rMM:Ro2=(b1rΛs+crΛor)/J Rn2=(drΛs+Λor)/J

where *J* is the determinant

(11) $$J=b_{1r}-c_{r}d_{r}$$

which we assume to be nonzero. The MM solution is a quasiperiodic solution of (6) since two frequencies are nonzero, as can be seen from the two phase equations

$$\begin{array}{l} \phi_{A,T}=c_{i}(r_{C}^{2}-r_{D}^{2}),\\ \phi_{C,T}=\Lambda_{oi}-b_{1i}r_{C}^{2}-b_{2i}r_{D}^{2}+d_{i}(r_{A}^{2}+r_{B}^{2}) \end{array}$$

The OS-solution exists in Λ*_s_* *>* 0 and encounters a transition to instability along the half-line

(12) $$(\text{O}):\;\;\;d_{r}\Lambda_{s}+\Lambda_{or}=0,\;\;\;J(b_{1r}\Lambda_{s}+c_{r}\Lambda_{or})\geq 0$$

Likewise the NT-solution exists in Λ*_or_* *>* 0 and encounters a transition to instability on the half-line

(13) $$(\text{N}):\;\;\;b_{1r}\Lambda_{s}+c_{r}\Lambda_{or}-0,\;\;\;J(d_{r}\Lambda_{s}+\Lambda_{or})\geq 0$$

The two half-lines (O) and (N) define a wedge in the (Λ*_s_,* Λ*_or_*)-plane, and in this wedge the MM-solution exists. Moreover, MM is stable (unstable) if *J >* 0 (*J <* 0).

**Figure 7:**
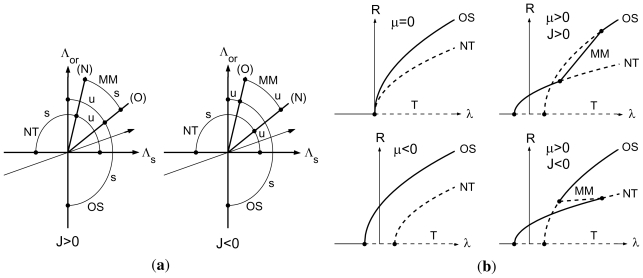
(**a**) Stability diagrams showing existence and stability regions (s: stable, u: unstable) of OS, NT, and MM in the (Λ*_s_,* Λ*_or_*)-plane in the case of (8), (9), and *d_r_* *<* 0, *c_r_* *<* 0. The straight arrow pointing from the third to the first quadrant corresponds to the path traversed for *μ* = 0 when *λ* increases from negative to positive values and (14) holds. (**b**) Sketch of bifurcation diagrams of OS, NT, and MM for fixed *μ* = 0, *μ <* 0 and *μ >* 0 with *J <* 0 and *J >* 0 in the case of (8), (9), (14), (15) and *b*_1_*_r_* *> a_or_*. Stable and unstable branches are marked solid and dashed, respectively.

In Figure 7(a), the information about the existence and stability of the three solutions (10) is summarized in the form of stability diagrams in the (Λ*_s_,* Λ*_or_*)-plane for the two cases *J >* 0 and *J <* 0, and with both *c_r_* *<* 0 and *d_r_* *<* 0. The existence domains of the three solutions are indicated by circle-segments, and the dots on these segments separate regions in which the solution is stable (s) and unstable (u). The trivial solution is stable only in the third quadrant. The straight arrow pointing from the third quadrant to the first quadrant indicates the path traversed for *μ* = 0 (*ω*_0_ = *ω*_0_*_c_*) when *λ* increases from negative (*V < V_c_*) to positive (*V > V_c_*) values, thereby crossing the mode-interaction point at the origin. The primary bifurcations occur on the axes, and the secondary bifurcations on the half-lines (O) and (N) defined by (12) and (13). If *d_r_* *>* 0 and *c_r_* *>* 0, (O) and (N) are located in the fourth and second quadrant, respectively.

The path for *μ* = 0 is below the wedge in which MM exists, as indicated in Figure 7(a), if

(14) $$\begin{array}{l} J>0,\;d_{r}<0,\;0<a_{or}<-d_{r}\\ \text{or }\;\;J<0,\;c_{r}<0,\;0<a_{or}<-b_{1r}/c_{r} \end{array}$$

The bifurcation diagram *R* versus *λ* for this path is sketched in the upper left panel of Figure 7(b). The other three bifurcation diagrams in this figure are for *μ <* 0 and *μ >* 0 with *J >* 0 and *J <* 0, and

(15) $$a_{or}b_{s}-b_{or}>0$$

Which implies that the path in Figure 7(a) is translated upward for *μ >* 0 and downward for *μ <* 0. In addition it is assumed that *b*_1_*_r_* *> a_or_* in all four diagrams, leading to an intersection of the OS and NT branches for *μ >* 0 (which does not correspond to a stability exchange). In all four bifurcation diagrams stable branches are displayed solid and unstable branches dashed.

Stability exchanges of the primary branches occur when the perturbed path for *μ >* 0 crosses the (O) and (N) half-lines. These stability exchanges are combined with secondary bifurcations of MM, which connects the two bifurcation diagrams as indicated in the right panels of Figure 7(b). The amplitude *R* along MM is $R=\sqrt{R_{o}^{2}+R_{n}^{2}}$, and the MM-branch has been sketched as straight line for simplicity. The bifurcation scenarios of Figure 7 are consistent with our experimental observation that in the NT-regime (*ω*_0_ *> ω*_0_*_c_*) the OS-rolls reappear when *V* is further increased above the *V_oc_*-threshold. Note that *J >* 0 leads to a continuous transition of stable branches, NT→MM→OS, whereas for *J <* 0 we find bistability leading to hysteresis.

In summary, consistency with our experiments requires that the conditions (8), (9), (14), and (15) are satisfied by the normal form coefficients, but we cannot discriminate between *J >* 0 and *J <* 0. The global persistence of the OS-rolls is apparent from the clear OS-pattern in Figure 3(a) in the OS-regime *ω*_0_ *< ω*_0_*_c_*, whereas in Figure 3(b) we observe “almost” pure NT-rolls with small patches of OS-rolls. Such patches cannot be explained on the basis of the normal form (6), but require numerical studies of the Ginzburg–Landau system (2).

In the normal form description, the critical voltages are given by Λ*_s_* = 0 and Λ*_or_* = 0, which leads to

$$\begin{array}{l} V_{sc}=V_{c}(1+b_{s}\mu ),\\ V_{oc}=V_{c}(1+(b_{or}/a_{or})\mu ) \end{array}$$

The threshold curves depicted in Figure 2(a) show that the stationary critical voltage, *V_sc_*, is strongly decreasing from *V_c_* when *μ* is decreasing in *μ <* 0, whereas the oscillatory critical voltage, *V_oc_*, is weakly increasing from *V_c_* in *μ >* 0. Thus we can conclude that the coefficients in Λ*_s_* and Λ*_or_* satisfy

$$0<b_{or}\ll a_{or}b_{s}.$$

## 4. Conclusions

In this paper we have presented and analyzed the first reported occurrence of near-onset patterns dominated by the interaction of steady oblique rolls and normal traveling rolls in nematic elctroconvection experiments. The results described in this paper confirm that nematic electroconvection is a multi-parameter physical system that naturally exhibits this kind of mode interaction. In addition, our experiments also confirm the weak electrolyte model as the correct theoretical description governing the spatiotemporal dynamics of nematic elctroconvection, since it predicts oblique as well as normal rolls at the onset and both types of rolls can be stationary or traveling. As common in spatially extended systems, we did not observe ideal roll patterns, but patches of ideal patterns separated by domain walls.

A pivotal result of our qualitative theoretical study is the derivation of the system of globally coupled Ginzburg–Landau equations governing the dynamics of slowly varying spatiotemporal envelopes of ideal roll patterns in anisotropic systems near the experimentally observed codimension-two point. We have identified primary solution branches, studied their stability, and identified regions in parameter space giving rise to superpositions of these solutions (mixed mode solutions) in the context of an idealized normal form description restricted to spatially uniform envelopes of ideal patterns. The main features of the resulting bifurcation diagrams are that there is either a continuous transition between the two primary branches via a stable mixed mode branch, or a region with bistability and an unstable mixed mode branch leading to a hysteretic transition. Our experiments do not yet provide evidence which of the two scenarios is present in the physical system. Further experiments in which the voltage is carefully increased and decreased for *ω*_0_ *> ω*_0_*_c_* combined with a thorough analysis of the recorded patterns are necessary to discriminate between the two scenarios.

The next step in the theoretical analysis of the mode interaction will be a numerical study of the patterns predicted by the globally coupled Ginzburg–Landau equations. Of special interest here is the region in which the normal traveling waves are created in the primary instability. We expect that the two normal form scenarios described above will lead to rather different spatiotemporal patterns, which will provide further criteria allowing to distinguish between them in experiments. Ultimately, the connection between the experiment and the theoretical model has to be established by computing the coefficients of the Ginzburg–Landau equations from the equations of the weak electrolyte model for the material parameters of the Phase V sample used in the experiments. Such calculations have been performed in [16] for the case of the oblique oscillatory instability in the nematic liquid crystal I52.
